# Comparative analysis of different types of occlusal splints for the management of sleep bruxism: a systematic review

**DOI:** 10.1186/s12903-023-03782-6

**Published:** 2024-01-05

**Authors:** Sultan Ainoosah, Ahmed E. Farghal, Marwa Saad Alzemei, Ravinder S. Saini, Vishwanath Gurumurthy, Syed Altafuddin Quadri, Abdulmajeed Okshah, Seyed Ali Mosaddad, Artak Heboyan

**Affiliations:** 1 Department of Substitute Dental Science, College of Dentistry, Taibah University, Madinah, Saudi Arabia; 2 Department of Restorative Dental Sciences, College of Dentistry, Taibah University, Madinah, Saudi Arabia; 3 Department of Dental Technology, COAMS, King Khalid University, Abha, Saudi Arabia; 4 Student Research Committee, School of Dentistry, Shiraz University of Medical Sciences, Qasr-E-Dasht Street, Shiraz, Iran; 5 Department of Prosthodontics, Faculty of Stomatology, Yerevan State Medical University after Mkhitar Heratsi, Str. Koryun 2, Yerevan, 0025 Armenia

**Keywords:** Occlusal splint, Sleep bruxism, Soft splints, Hard splints, Adjustable splints

## Abstract

**Background:**

Sleep bruxism is a prevalent condition in dentistry practice, characterized by involuntary grinding or clenching of the teeth during sleep. Several therapies, including occlusal splints, have been used to manage sleep bruxism and temporomandibular disorders, including occlusal splints. This study aimed to compare the effectiveness of different occlusal splints in managing sleep bruxism.

**Methods:**

The PICO framework encompasses the characterization of the population, intervention, comparison, and pertinent outcomes. A comprehensive and systematic literature review was conducted on PubMed, Scopus, and Google Scholar to identify grey literature. The search specifically targeted scientific studies published before September 20, 2023. The Cochrane Collaboration Risk of Bias Tool assessed the accuracy of the included Randomized Control Trials (RCTs). The modified Newcastle–Ottawa Scale assessed non-randomized studies. Data were systematically extracted, synthesized, and reported thematically.

**Results:**

Out of the total of 808 articles that were evaluated, only 15 articles were found to meet the specified inclusion criteria. Adjustable splints, such as full-occlusion biofeedback splints, were more effective in reducing sleep bruxism episodes, improving patient-reported symptoms, and enhancing overall well-being. The impact of different occlusal sprints on electromyographic activity varies, and potential adverse effects should be considered individually.

**Conclusions:**

This review provides valuable insights into the effectiveness of occlusal splints in managing sleep bruxism. The results of this study indicate that occlusal splint therapy is a viable treatment approach for sleep bruxism.

## Background

Temporomandibular disorders (TMD) are a group of conditions that cause pain and dysfunction in the temporomandibular joint (TMJ) and surrounding muscles [1]. TMDs are often associated with biomechanical issues such as malocclusion, in which the teeth are not properly aligned when the jaws are closed. TMD can also be caused by bruxism (long-term teeth grinding or clenching) and abnormal jaw growth patterns in children [2]. Sleep bruxism is commonly characterized by rhythmic or non-rhythmic grinding or clenching of the teeth while asleep [2, 3]. In otherwise healthy individuals, it is not considered a disorder but may be a risk factor for negative oral health consequences like TMD [4–6]. In the study by Dias et al. [7], sleep bruxism was reported with a 67.6% occurrence of degenerative changes in the temporomandibular joint. These changes lead to pain, stiffness, reduced range of motion, and clicking or popping sounds in the jaw [7–9]. Sleep bruxism is also reported to affect sleep patterns and the general quality of life [3, 10].

Several therapies, including occlusal splints, have been used to manage sleep bruxism and temporomandibular disorders, including occlusal splints [11]. Occlusal splints, often night guards, are dental appliances that function as protective barriers. Their primary purpose was to minimize the detrimental consequences of bruxism by obstructing tooth-to-tooth contact during sleep [11]. Various occlusal splints are available to accommodate individuals' specific requirements and preferences [12].

Occlusal or bite splints, used for the management of TMD, are typically made from materials like acrylic [11]. The splints provide a barrier between dental arches to minimize the impact of temporomandibular disorders. Since these disorders have adverse symptoms like pain, the cushioning effect of the splints helps improve comfort and minimizes accompanying symptoms [11, 13]. Splints are customized to suit the different needs of patients by taking impressions of patients' teeth, which are used to fabricate customized splints for specific bite patterns. Splints customization ensures optimal comfort and compliance [14].

Traditional acrylic splints are known to exhibit durability and can endure the stresses exerted during bruxism episodes [15]. Soft splints are often fabricated using soft substances such as silicone and are renowned for their exceptional comfort [16]. These splints provide a cushioning mechanism that mitigates the impact of clenching and grinding on the teeth and jaws [16]. There has been growing interest in using digital splints that include materials such as Polyether Ether Ketone (PEEK), owing to their precise nature and the ability to customize them according to individual patients [15]. The production of these splints utilizes sophisticated digital technology that guarantees a customized fit that maximizes both comfort and efficacy [15].

Besides occlusal splints, there are other methods employed in TMD management. These include massaging muscles surrounding the jaw, Botox Injections, transcutaneous electrical nerve stimulation (TENS), acupuncture, and low-level laser therapy (LLLT) [17–21]. Additionally, biofeedback has been used to manage TMD to help patients learn to control their masticatory muscle activity and reduce pain [22, 23]. Biofeedback can also help promote relaxation and regulate muscle tension [22, 24].

The rationale for conducting this systematic review was based on the need to assess and compare different occlusal splints used to treat sleep bruxism [12]. Sleep bruxism has oral health implications, including tooth wear, fractures, and orofacial discomfort, which require effective interventions [10]. The utilization of occlusal splints has emerged as a significant treatment modality. Nevertheless, various splints exist, each possessing distinct benefits and drawbacks [25]. This review aimed to evaluate and compare the efficacy of several occlusal splints in managing sleep bruxism. This would provide valuable insights for clinical practitioners and contribute to evidence-based decision-making in treating sleep bruxism.

The main objective of this systematic review was to comprehensively evaluate and consolidate the current body of literature to compare the effectiveness of different occlusal splints in treating sleep bruxism. It further aims to address the following research questions:

i. What is the impact of different occlusal splints on reducing sleep bruxism episodes and improving symptoms?
ii. How do various occlusal splints influence changes in electromyographic activity and patient-reported outcomes?

## Methods

The Preferred Reporting Items for Systematic Review and Meta-Analysis (PRISMA) guidelines [26] were used to identify published studies that explored and compared different occlusal splints for managing sleep bruxism. The protocol used for this systematic review was the registered International Platform of Registered Systematic Review and Meta-Analysis Protocols (INPLASY) (20231000489). To examine the existing information on various types of occlusal splints, the PICO framework [27] was defined as follows:

i. Population: Patients diagnosed with sleep bruxism.
ii. Intervention: Usage of occlusal splints to treat sleep bruxism.
iii. Comparison: Comparison between different types of occlusal splints.
iv. Outcome: Effectiveness of occlusal splints in managing sleep bruxism.

### The methodology employed for the comprehensive search of relevant literature

A detailed systematic literature search of PubMed, Scopus, Embase, and Google Scholar for gray literature was performed for articles published before September 20, 2023. A search was conducted to identify scholarly articles that compared different types of occlusal splints for managing sleep bruxism. The following keywords were applied: ("Sleep bruxism" OR "Nocturnal teeth grinding" OR " teeth clenching") AND ("Occlusal splints "OR "Night guards" OR "Dental appliances" OR "Hard splints" OR "soft splints" OR "Occlusal splint"). The process involved the consideration of synonyms and alternative spellings. The search terms were extensively employed in numerous combinations across multiple databases.

### Inclusion and exclusion criteria

Eligible studies included in this review met the following inclusion criteria: published in peer-reviewed journals; available in English; Studies that evaluated the effectiveness of different types of occlusal splints for the management of sleep bruxism; Studies conducted on human participants diagnosed with sleep bruxism; and studies that had clearly defined occlusal splint types and protocols for their use. Studies that did not meet the eligibility criteria were excluded from the analysis. These exclusions were based on the following reasons: the studies were in the form of reviews, meta-analyses, abstracts, or editorial articles; they lacked detailed descriptions of their methodology or results; they did not specifically investigate the comparison of various types of occlusal splints for the management of sleep bruxism; and they primarily focused on other interventions for sleep bruxism management, such as medication or behavioral therapies.

### Study selection

After completing the initial search strategy, articles were selected using a systematic step-by-step procedure. The Zotero Reference Manager was utilized to eliminate duplicate research, including the remaining studies in the subsequent screening steps. A comprehensive review was conducted on all possible articles. The titles and abstracts underwent an initial screening process to exclude research that did not meet the eligibility criteria. The complete texts of the remaining articles were assessed using the predetermined criteria for inclusion and exclusion. The relevancy of articles that satisfied all the inclusion criteria was evaluated.

### Risk of bias assessment

Eligible studies were critically checked for quality. The Cochrane Collaboration Risk of Bias Tool assessed the accuracy of the included Randomized Control Trials (RCTs) [28]. The modified Newcastle–Ottawa Scale assessed non-randomized studies [29].

### Data analysis

The data from the research included in this analysis were systematically collected and summarized in Table 1. The results were thematically reported according to the prevalence and clinical presentation, electromyographic (EMG) activity changes and sleep bruxism episodes measured using polysomnography (PSG), BiteStrip®, and advanced EMG analysis. Quantitative data were analyzed using Review Manager version 5.4.1.

**Table 1:** Summary of study characteristics and outcomes [5, 12–25]

Authors	Study Design	Sample Size	Study Region	Participants Characteristics	Details of occlusal splints evaluated	Outcome measure	Major Findings
Wang et al., [15]	RCT	18	China	Age range (18–44)	The test group consisted of digital splints that were planned and manufactured using computer-aided design and computer-aided manufacturing (CAD/CAM) techniques. In contrast, the control group consisted of rigid splints constructed using transparent acrylic resin.	Compared with the control group, the manual time spent in the test group was significantly less. No statistically significant differences were observed across the groups across the VAS retention scores. However, the test group exhibited significantly higher scores regarding wearing comfort. The test group showed significantly lower levels of both maximum depth loss and volumetric loss compared to the control group.	Compared to conventional rigid splints, the digitally generated splints were associated with better comfort and time efficiency. The new milling material (PEEK) also offers superior wear resistance to acrylic resins.
Karakis et al., [30]	RCT	12	Turkey	Students with Age range (18 to 27)	Rigid stabilization splints fabricated from auto-polymerizing acrylic resin. v/s Bruxogard-soft splints are prepared easily without impression.	Using rigid stabilization splints yielded no statistically significant alterations in occlusal force. The Bruxogard-soft splint demonstrated a statistically significant reduction in occlusal force. A statistically significant improvement was observed in the individuals' CMI value in both groups utilizing splints.	Participants' utilization of a Bruxogard-soft splint resulted in a reduction in occlusal force. Utilizing both splints resulted in a notable decrease in the clinical manifestations.
Harada et al., [31]	RCT	16	Japan	Not reported	Stabilization splint (SS) v/s palatal splint (PS). Both are rigid splints made of heat-cured hard acrylic with a wax-up method.	Both splints demonstrated a considerable decrease in SB immediately upon device insertion; however, no further reduction was observed. No statistically significant difference was observed in the impact on SB between the SS and PS conditions.	Both splints demonstrated a reduction in masseter electromyography (EMG) activity that was related to sleep bruxism (SB); however, it should be noted that this impact was temporary.
Dalewski et al., [32]	RCT	30	Poland	mean age 24.8	Okeson's Stabilization Splint is mainly made for the upper arch to position the mandible (lower jaw) in a stable musculoskeletal position. v/s Bimaxillary Involves both the upper and lower arches. It positions the mandible in a stable musculoskeletal position.	In both groups, pain was significantly reduced.	Both splint designs can effectively alter the pressure pain threshold in patients with diagnosed bruxism.
Dubé et al., [33]	RCT	9	Canada	5 Females 4 Males (mean age = 23.7)	Hard Acrylic Occlusal Splint- made of hard acrylic material and has a U-shaped design. It is designed to be worn on the maxillary (upper) arch and adjusted to the patient's centric tooth relation—v/s Palatal Control Device –inserted on the maxillary arch and modified for maximum tooth intercuspation.	The two devices demonstrated a statistically significant decrease in the frequency of SB episodes per hour and SB bursts per hour. Both oral devices showed a reduction of 50% in the occurrence of grinding noise episodes. There was no discernible distinction noticed between the devices. Both devices demonstrated a decrease in muscle activity related to sedentary behavior.	Oral devices minimize SB promotor episodes and tooth-grinding in SB patients.
Okeson, [34]	Intraparticipant design	10	Not reported	Females = 5; males = 5. Mean Age = 27.4 years	Hard Acrylic Occlusal Splint- Covers all maxillary teeth. It is adjusted for even and simultaneous contacts of mandibular buccal cusps and incisal edges in centric relation. v/s Soft Vinyl Occlusal Splint—Made from thick, soft vinyl sheets adapted to the maxillary cast. Adjusted to achieve even contact of mandibular teeth during light closure.	The rigid occlusal splint significantly reduced muscular activity among 80% of the individuals (8 out of 10). Utilizing the soft occlusal splint resulted in a notable decrease in muscle activity in one person while inducing a statistically significant elevation in muscle activity in five out of the ten participants.	A soft occlusal splint may not be recommended in individuals exhibiting symptoms linked to heightened muscular activity throughout the night. A rigid occlusal splint seems to be a more probable practical course of therapy.
de Paula Gomes et al., [35]	RCT	60	Brazil	Age range (18–40)	A Michigan-type occlusal splint—molded for each volunteer's upper arch containing canine and protrusive guides and a flat occlusal surface for contact with the teeth. v/s The silicone occlusal splint- is made by vacuum pressure molding from a 3-mm soft polyvinyl sheet.	Either massage treatment or an occlusal brace did not significantly alter the electromyographic activity of the masseter and anterior temporal muscles.	A multimodal approach may be more effective in managing the complex presentation of TMD and sleep bruxism.
Bergmann et al., [36]	RCT	41	Germany	females = 21; males = 18. Mean Age = 41.3 ± 14.2(control) 37.6 ± 11 (test)	The Adjustable Occlusal Splint (AOS) comprises transparent auto-polymerizing dental acrylic resin. It exhibits a level occlusal surface with consistent contact points in the centric relation and anterior guidance for movements during excursive actions. versus The Biofeedback Splint (BFB) consisted of soft thermoformed maxillary dental plates that provided a full covering.	The biofeedback group improved the patient's overall well-being, face muscle pain, and decreased burst frequency and length. The average and maximum durations in the biofeedback group were statistically reduced after the therapy was terminated.	Biofeedback splint improves the patient's pain perception and lowers SB. The findings imply that an AOS (adjusted occlusal splint) is not the most effective therapy for discomfort associated with bruxism compared to the biofeedback splint.
Lei et al., [37]	Pre-post Intervention Analysis	16	China	Age Range 20 to 45 yrs	The occlusal splint is composed of a thermoplastic material with a thickness of 1.5 mm. versus. The occlusal splint is composed of a soft thermoplastic material with a thickness of 1.5 mm. A revised anterior splint design, which comprises a thermoplastic partial rigid occlusal splint with a thickness of 1.5 mm, is proposed. This splint is intended to cover the anterior maxillary teeth. A flat bite plate made of self-curing resin is also on the palatal side.	The individuals who used a modified anterior splint had substantially lower EMG data than those who used a hard or soft occlusal splint or did not use a splint. Participants who do not use a splint exhibit the highest biting force and bite area, while participants who utilize a modified anterior splint have the lowest bite force and bite area.	The utilization of a modified anterior splint has been found to exhibit enhanced comfort and efficacy in mitigating occlusion force and electromyographic activity of the anterior temporalis and masseter muscles among individuals with bruxism.
Kolcakoglu et al., [38]	RCT	240	Turkey	children with a Mean Age of 8.6 yrs	Soft Occlusal Splints- made using a 1.5 mm-thick soft thermoplastic material designed to cover all teeth' incisal and occlusal surfaces in a U-shape. v/s Rigid Occlusal Splints—made using a 1.5 mm-thick thermoplastic hard material and covered all teeth' incisal and occlusal surfaces in a U-shape like the soft splints	Using a soft occlusal splint resulted in a considerable reduction of muscle discomfort during palpation and pain experienced in the dynamic position of the temporomandibular joint (TMJ) pain among patients. The BiteStrip® score for groups I and II showed no statistically significant alteration.	Soft occlusal splints have the potential to alleviate discomfort resulting from nocturnal bruxism, particularly about the muscles and temporomandibular joint (TMJ). No significant correlation exists between treatment outcomes and BiteStrip® ratings among patients using soft or hard occlusal splints.
Ariji et al., [39]	RCT	16	Japan	Men = 11 women = 5; Age Range 27–53 yrs. Mean Age 35.5 yrs	Soft Splints were customized for each subject and made from 3-mm ethylene–vinyl acetate sheets with a hardness of 7955g. v/s Rigid Splints were customized for each subject and made from 3-mm polyethylene terephthalate glycol sheets. The splints had a flexural strength of at least 55 MPa and a flexural modulus of around 2000 MPa.	The rigid splint resulted in an augmentation of the blood oxygen level-dependent (BOLD) signals inside BA6 and BA20. The blood oxygenation level-dependent signals in the left BA6, the left BA20, 37, and the right BA44, 45 exhibited a statistically significant increase when clenching with a rigid splint compared to natural teeth.	Clenching one's jaw while wearing a rigid splint resulted in neural activation throughout many expansive brain areas, including the specific area connected with the coordination of motor functions.
Lukic et al., [40]	RCT	10	Switzerland	Males = 4 females = 6, Mean Age = 30 ± 6 yrs.)	Michigan Splint: Customized for each participant using (CAD/CAM) and manufactured from methyl Methacrylate, a hard-acrylic resin. v/s NTI-tss Device -Designed to cover the maxillary incisors and contact only the tips of the lower middle incisors and filled chair-side with auto-polymerizing acrylate to adjust the patient's teeth	The NTI-tss device was associated with a reduction in muscle activation. The Michigan splint was shown to be the favored choice among most patients, primarily because of its superior level of wearing comfort.	NTI-tss devices have shown more efficacy in reducing jaw muscle activation during sleep. One notable benefit of the prefabricated NTI-tss is its immediate accessibility during the acute phase of temporomandibular disorders linked to sleep bruxism. In addition, it is essential to consider subjective preferences, the level of wearing comfort, and the associated expenditures.
Al Quran & Lyons, [41]	Not Clear	10	UK	Young adults (age range 21–34 yrs.)	Rigid Acrylic Resin Splint- Made to a jaw registration with the mandible in the (retruded) *returned* position, anterior teeth approximately 2 mm apart, and widespread occlusal contacts in the (retruded returned position. v/s Soft Acrylic Resin Splint: Vacuum-formed on a cast of the upper teeth with no occlusal adjustment.	The study revealed that using rigid splints reduced electromyographic (EMG) activity compared to the absence of splints during maximal clenching. This effect was seen in both muscles, with a notable drop in the anterior temporalis muscle. The use of soft splints resulted in a modest elevation in the activity of both muscles, with a specific emphasis on the masseter muscle.	The therapeutic impact of both a hard and a soft splint may be attributed, in part, to a reduction in the activity of the temporalis muscles compared to the masseter muscles. However, it is worth noting that this decrease is more pronounced with a rigid splint.
Deregibus, [42]	RCT	40	Italy	13 Males 27 Females 47.2 ± 12.8 yrs.; range, 22 to 56 yrs.)	Upper Michigan OS (Group 1)- in contact with the mandibular supporting cusps and had cuspid guidance that disclosed the supporting cusp contact almost as soon as lateral movements were made. v/s Mandibular OS (Group 2)- constructed to allow only posterior contacts (from the second premolar to the second/first permanent molar) without static and dynamic anterior contacts.	The outcome measures tested within both groups showed no statistically significant differences. Nevertheless, it was observed that Group 2 exhibited a notably more excellent range of motion (ROM) in the right lateral mandibular direction at T2 and a substantially higher ROM in the left lateral mandibular direction during T3.	The research findings indicate that an orthodontic splint (OS), regardless of its placement on the upper or lower arch, does not substantially impact pain reduction among patients with temporomandibular disorder (TMD) during six months.
Silva et al., [43]	Not Clear	1	Brazil	Young-adult male patient	Hard v/s soft Occlusal Splint (OS)- A standard OS with a 3 mm thickness, designed for the study subject, was used during image acquisition and later created as a part of the 3D model.	Comparing occlusal splints showed no significant stress intensity or distribution changes in the left or right TMJ discs. It should be observed that the anterior disc was most stressed.	Using rigid acrylic occlusal splints over soft ones is advisable in most situations. It is recommended to prioritize using thinner operating systems, with an anterior thickness ranging from 2 to 3 mm, as opposed to thicker ones measuring 3 to 4 mm. It is advisable to choose lighter contacts over heavy contacts when considering the contact surface area of the second molar to mitigate stress concentrations and minimize the risk of fractures.
TOTAL		529					

## Results

### Study selection process

**Fig. 1:**
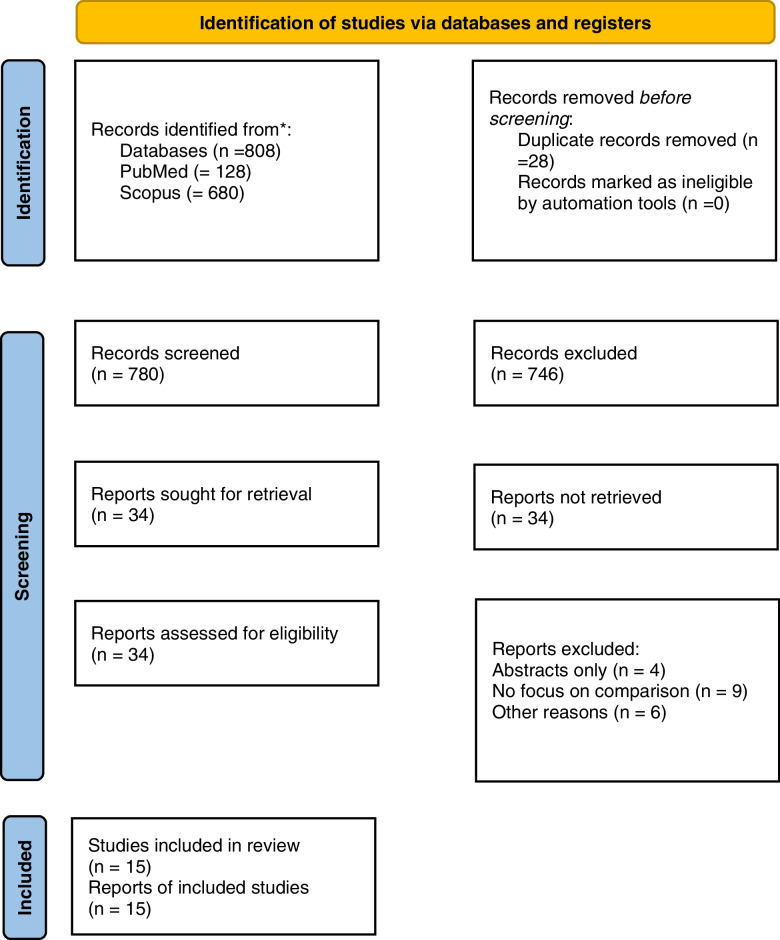
PRISMA flow diagram for Systematic review with included searches of databases

The database search resulted in 808 articles, from which 28 duplicate entries were subsequently eliminated. A total of 746 articles were eliminated through title and abstract screening. Thirty-four publications were obtained and evaluated to determine their suitability for inclusion in this study. A total of nineteen papers were excluded from the study due to their failure to satisfy the predetermined inclusion criteria. Fifteen publications were finally deemed appropriate for review after screening, as depicted in Fig. 1.

### Methodological quality assessment

**Fig. 2:**
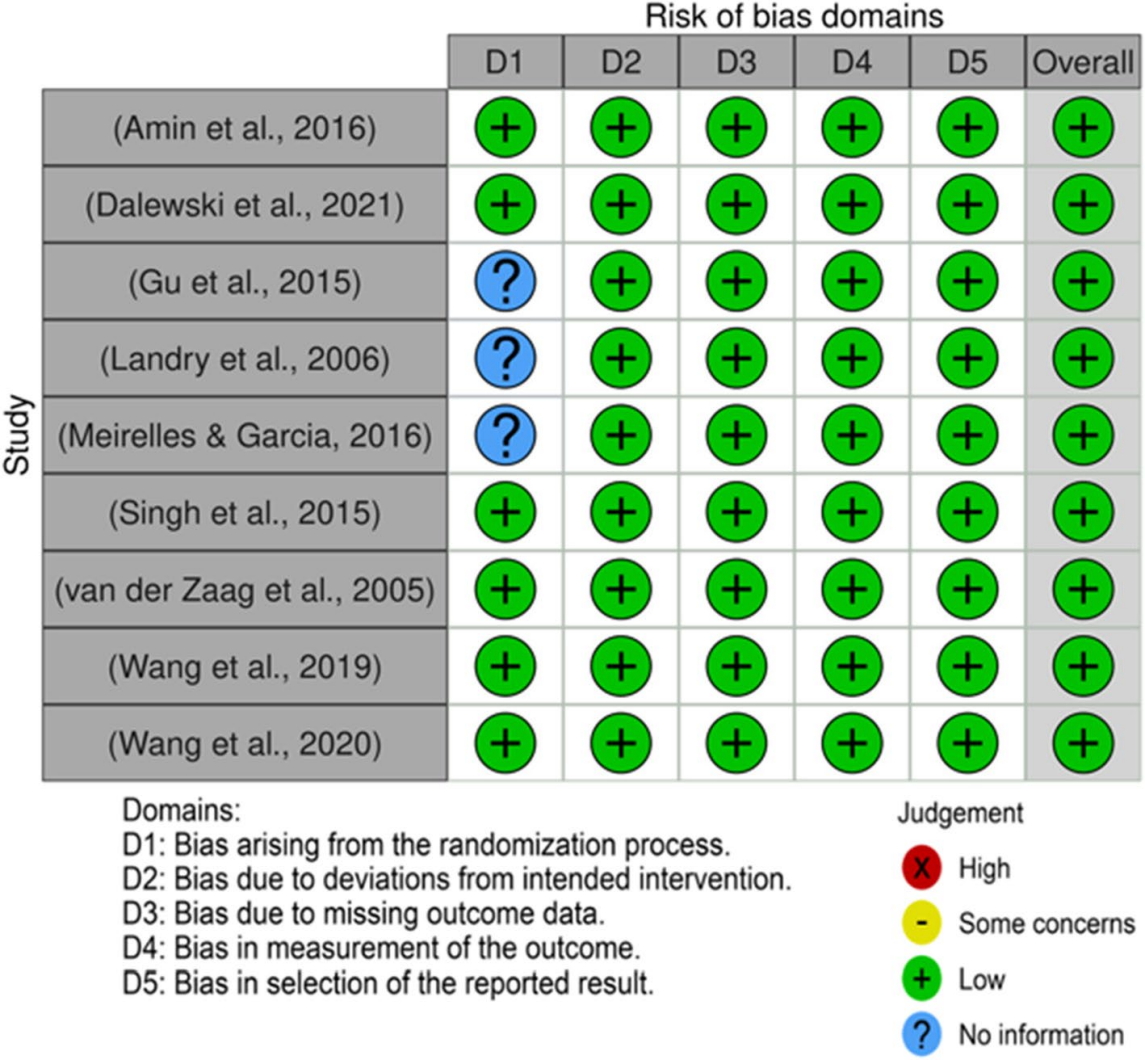
The critical evaluation of the studies is represented by a traffic light plot [5, 12–21]

**Fig. 3:**
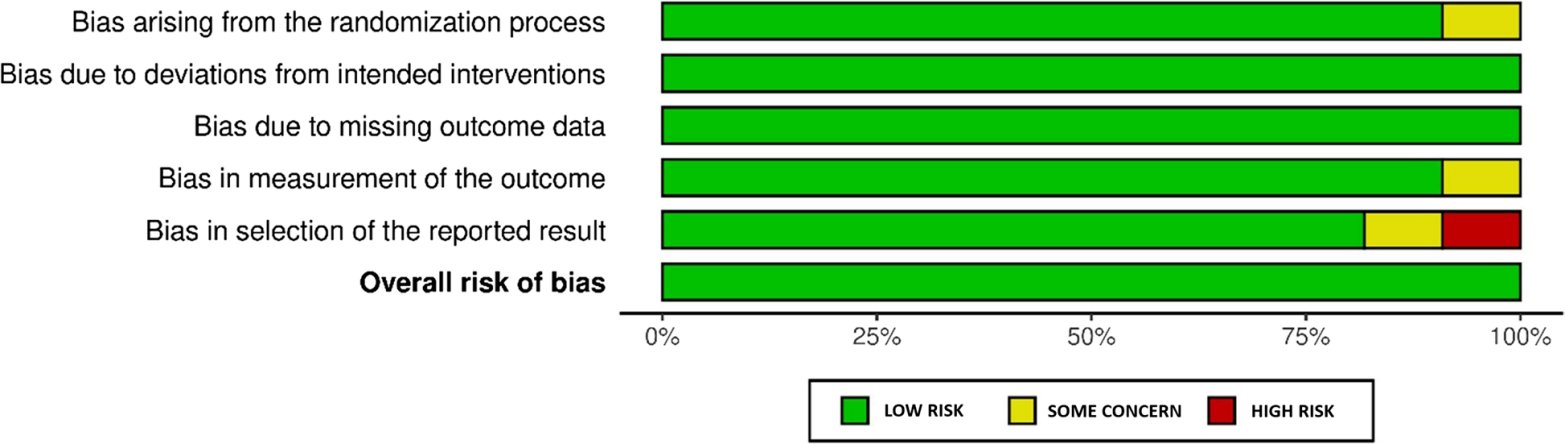
Graph summarizing the findings from the studies' critical analyses

The outcomes were presented in traffic light plots and summary plots for critical appraisal of the studies, as shown in Figs. 2 and 3.

### Main study characteristics

Table 1 provides a summary of the study characteristics and outcomes. These studies were published between 1987 and 2023. The study comprised a sample size of 529 people. The majority of the research included in the analysis were RCTs. The participants were predominantly young adults with an age range of 18–40 years. Most studies have been conducted in Asian countries. Occlusal splints identified could be categorized as soft, complex, and adjustable.

### Comparative analysis of different occlusal splint types

Occlusal splints have evolved as a multifaceted therapeutic intervention for various illnesses, including temporomandibular disorders and sleep bruxism. Multiple types of occlusal splints have different effects on managing sleep bruxism. The effectiveness of soft, rigid, and adjustable splints was analyzed using thematic analysis [30]. The dominant impacts of different occlusal splints were categorized into the following four groups:

### Reduction in sleep bruxism episodes

Some studies have assessed the impact of different occlusal splints on sleep bruxism episodes and demonstrated mixed results in mitigating the frequency and severity of sleep bruxism. Harada et al. [31] examined the impact of stabilization and palatal splints on sleep bruxism. Both splints showed reduced bruxism immediately after insertion. However, this impact was temporary and did not persist beyond 2, 4, or 6 weeks [31]. Additionally, there was no statistically significant difference in the effect of a stabilization splint and palatal splint on sleep bruxism. Similarly, Dubé et al. [33] compared the effectiveness and safety of an occlusal splint and a palatal control device in individuals diagnosed with sleep bruxism. Both devices significantly reduced the frequency of sleep bruxism episodes per hour. Notably, no statistically significant difference in efficacy was observed between the two devices [33].

In contrast, Bergmann et al. [36] evaluated the efficacy of a full-occlusion biofeedback splint compared to an adjusted occlusal splint in treating sleep bruxism. The full-occlusion biofeedback splint group showed a decreased frequency and duration of bruxing events. After treatment cessation, the biofeedback group maintained a reduction in the burst duration. This suggests that the biofeedback splint effectively reduced sleep bruxism at the subconscious level and improved pain perception [36].

### Changes in Electromyography (EMG) activity

Electromyographic recordings of masticatory muscles may provide valuable insights into the efficacy of different occlusal splints [11]. Lei et al. [37] investigated the effects of two occlusal splints that provide full coverage and a modified anterior splint on persons diagnosed with bruxism. The revised anterior splint demonstrated enhanced comfort and efficacy in reducing the occlusion force and electromyographic activity of the anterior temporalis and masseter muscles [37]. Conversely, Okeson [34] examined the effects of hard and soft occlusal splints on nocturnal muscle activity. The findings revealed that using rigid occlusal splints substantially reduced muscular activity in eight subjects. However, soft occlusal splints have contrasting outcomes. The procedure resulted in a significant decrease in muscle activity in one individual while conversely resulting in a statistically significant increase in muscle activity in five participants [34].

Moreover, de Paula Gomes et al. [35] investigated the effects of various treatments, including massage therapy, conventional occlusal splint therapy, and silicone occlusal splint therapy, on electromyographic activity in the masseter and anterior temporal muscles of individuals with sleep bruxism. Surprisingly, their findings demonstrated that neither massage nor occlusal splint therapy significantly impacted electromyographic activity. However, an intriguing discovery emerged when both therapeutic approaches were used in conjunction; there was a notable decrease in the severity of clinical manifestations observed in patients who were diagnosed with both severe temporomandibular disorder and sleep bruxism. This finding may provide promising insights for patients diagnosed with severe temporomandibular disorder and sleep bruxism, as the use of both therapeutic approaches simultaneously could potentially lead to improved outcomes.

### Improvement in symptoms

Certain studies have investigated the symptom burden in persons with bruxism using different occlusal splints. The effectiveness of different occlusal splints in improving symptoms differed. De Paula Gomes et al. [35] examined the impact of several therapeutic interventions on the severity of clinical manifestations in patients diagnosed with sleep bruxism. They revealed that integrating massage, conventional occlusal splint, and silicone occlusal splint therapies decreased the severity of the patient's signs and symptoms. Bergmann et al. [36] showed that using a biofeedback splint increased patients' overall well-being and comfort.

Kolcakoglu et al. [38] investigated the efficacy of occlusal splint interventions in pediatric patients with nocturnal bruxism. The study discovered soft occlusal splints significantly reduced muscle discomfort and temporomandibular joint pain during palpation. Nevertheless, no statistically significant alteration was observed in the BiteStrip® score for either the soft or hard splint groups [38]. Dalewski et al. [32] assessed the efficacy of two distinct occlusal devices in individuals displaying symptoms indicative of bruxism. A comparable decrease in the pain factor was observed in both groups, irrespective of the gadget used.

### Patient-reported outcomes and adverse effects

Certain studies have assessed patient-reported outcomes and adverse effects and found that different occlusal splints had different effects on patient-reported outcomes and adverse effects. For instance, Deregibus [42] compared the effectiveness of upper Michigan occlusal splints and mandibular occlusal splints in individuals diagnosed with muscle-related temporomandibular disorders. The findings revealed no significant reduction in pain over six months. Nevertheless, the presence of mandibular occlusal splints was shown to correlate with enhanced jaw mobility during lateral movements at designated time intervals. This implies that while occlusal splints may not substantially affect pain reduction, they may have a limited influence on enhancing jaw mobility in individuals with temporomandibular disorders [42].

## Discussion

This systematic review presents a comparative examination of various occlusal splints utilized in managing sleep bruxism. This review demonstrated that adjustable splints, such as full-occlusion biofeedback splints, are more effective in reducing sleep bruxism episodes, improving patient-reported symptoms, and enhancing overall well-being. The impact of occlusal splints on electromyographic activity varies depending on the splint used and the individual's physiological reactions. Specific splints, such as the modified anterior splint, have shown greater efficacy in lowering muscle activity [37]. Conversely, soft occlusal splints have demonstrated varied outcomes. Therefore, the potential adverse effects should be considered on an individual basis. The selection of a splint should be well deliberated, considering individual requirements and preferences [44].

Critical analysis and synthesis of the results of multiple studies are essential for a comprehensive understanding of the effectiveness of occlusal splints in managing sleep bruxism. Bergmann et al. [36] showed that full-occlusion biofeedback splints effectively reduced sleep bruxism episodes. These results are consistent with previous research and the idea that splints may be an effective intervention to reduce the frequency of bruxism episodes during sleep [44].

Interestingly, other studies have shown no statistically significant differences in the impact of different splint devices on managing sleep bruxism [31, 33]. However, both splint devices significantly reduced the bruxism episodes upon their insertion. The lack of statistically significant differences may have been due to heterogeneity in the patient population. Bruxism is a complex condition influenced by stress, anxiety, and occlusal conditions. Including patients with varying degrees of bruxism may have affected their response to treatment.

De Paula Gomes et al. [35] and Kolcakoglu et al. [38] investigated sleep bruxism. They reported that improvement in the severity of signs and symptoms was associated with the use of combined therapies and soft occlusal splints. These findings highlight the possibility of integrating several therapies to mitigate the symptoms related to sleep bruxism and temporomandibular joint disorders [35]. Moreover, soft splints may more effectively mitigate symptoms in younger individuals diagnosed with bruxism [38]. In contrast, Dalewski et al. [32] discovered that Okeson's Stabilization Splint and Bimaxillary Splint had a comparable impact in reducing pain factors related to sleep bruxism. This implies that both splint designs can effectively alter the pressure pain threshold in patients diagnosed with bruxism [32].

Occlusal splints are also known to undergo changes over time. Their continuous contact with teeth during mastication and grinding leads to abrasion and changes in the splint's surfaces. The materials used in making both traditional and 3D-printed splints are also subject to aging. This is primarily due to exposure to oral fluids and temperature variations. Aging results in changes in the splints' mechanical and chemical properties, reducing their effectiveness [45]. The choice of the splint material thus plays a crucial role in determining splint longevity and performance. 3D-printed splints made of biocompatible polymers are preferred due to their proven stability in the oral environment [46, 47]. The better surface finish of 3D-printed splints also improves wear resistance and comfort.

### Strengths and limitations of included studies

Most of the included studies were RCTs. Randomization is critical for bolstering internal validity and eliminating selection bias. The use of patient-reported outcomes plays a crucial role in evaluating the efficacy of treatments from the standpoint of care recipients, thereby augmenting the comprehensiveness of the research results. The studies included in this review had certain limitations: heterogeneity in the types of occlusal splints and variability in outcome measures. While some studies utilized adjustable splints, others used hard or soft splints, making it difficult to compare the outcomes directly. Furthermore, some studies had short follow-up durations, limiting the capacity to evaluate occlusal splint treatment's long-term efficacy and stability. The article search was only restricted to research in English, limiting accessibility to studies in other settings published in other languages.

### Implications for clinical practice

Clinicians must acknowledge that occlusal splint treatment is valid for managing sleep bruxism and temporomandibular disorders [44]. Nevertheless, each patient's unique requirements and preferences must determine the selection of splint type. This review suggests that using a multimodal strategy that integrates occlusal splint treatment with complementary therapies, such as massage therapy, may result in more favorable outcomes, such as improved symptoms and overall well-being.

Patient-reported outcomes are paramount for assessing treatment efficacy [35]. Clinicians must prioritize patient-centered treatment by aggressively soliciting input from patients about their symptoms, degrees of pain, and general well-being [35]. Feedback should be used to make necessary changes and alterations to the treatment plan, prioritizing the patient's viewpoint within the care process.

### Recommendations for future research

Future advancements in occlusal splint treatment should place greater emphasis on patient-reported outcomes. This may be achieved using validated instruments to evaluate the extent to which symptoms are alleviated and the subsequent effects on patients' overall quality of life, sleep patterns, and general well-being. Adopting a patient-centered approach is crucial for adequately customizing therapies to meet individuals' unique requirements. Furthermore, future research endeavors should integrate sophisticated objective assessments such as advanced electromyographic analysis or polysomnography to understand the physiological alterations associated with occlusal splint therapy. These assessments should encompass various aspects, including muscle activity, sleep patterns, and the occurrence of bruxism. Further investigation of the side effects and tolerability is required, specifically focusing on examining the frequency and intensity of the adverse effects of occlusal splint treatment.

## Conclusion

This review provides valuable insights into the effectiveness of occlusal splints in managing sleep bruxism. Nevertheless, diverse therapies, limited follow-up durations, and inconsistent outcome measures across studies underscore the need for more extensive research in this field. The results of this study indicate that occlusal splint therapy is a viable treatment approach for sleep bruxism. However, further research is needed to understand its long-term effects, patient-reported outcomes, and adverse effects. The management of sleep bruxism requires careful consideration of several factors, highlighting the need to customize treatment approaches to suit the unique demands of each patient.

## Data Availability

The data supporting this study's findings are available from the corresponding author upon reasonable request.

## Abbreviations

PEEK: Polyether Ether Ketone
RCTs: Randomized Control Trials
EMG: Electromyographic
PSG: Polysomnography
